# Small Molecule-Based Promotion of PKCα-Mediated β-Catenin Degradation Suppresses the Proliferation of CRT-Positive Cancer Cells

**DOI:** 10.1371/journal.pone.0046697

**Published:** 2012-10-05

**Authors:** Jungsug Gwak, Jee-Hyun Lee, Young-Hwa Chung, Gyu-Yong Song, Sangtaek Oh

**Affiliations:** 1 Department of Advanced Fermentation Fusion Science and Technology, Kookmin University, Seoul, Republic of Korea; 2 PharmacoGenomics Research Center, Inje University, Busan, Republic of Korea; 3 College of Pharmacy, Chungnam National University, Daejeon, Republic of Korea; 4 WCU, Department of Cogno-Mechatronics Engineering, Pusan National University, Busan, Republic of Korea; Albert-Ludwigs-University, Germany

## Abstract

Aberrant accumulation of intracellular β-catenin is a well recognized characteristic of several cancers, including prostate, colon, and liver cancers, and is a potential target for development of anticancer therapeutics. Here, we used cell-based small molecule screening to identify CGK062 as an inhibitor of Wnt/β-catenin signaling. CGK062 promoted protein kinase Cα (PKCα)-mediated phosphorylation of β-catenin at Ser33/Ser37, marking it for proteasomal degradation. This reduced intracellular β-catenin levels and consequently antagonized β-catenin response transcription (CRT). Pharmacological inhibition or depletion of PKCα abrogated CGK062-mediated phosphorylation and degradation of β-catenin. In addition, CGK062 repressed the expression of the genes encoding cyclin D1, c-myc, and axin-2, β-catenin target genes, and thus inhibited the growth of CRT-positive cancer cells. Furthermore, treatment of nude mice bearing PC3 xenograft tumors with CGK062 at doses of 50 mg/kg and 100 mg/kg (i.p.) significantly suppressed tumor growth. Our findings suggest that CGK062 exerts its anticancer activity by promoting PKCα-mediated β-catenin phosphorylation/degradation. Therefore, CGK062 has significant therapeutic potential for the treatment of CRT-positive cancers.

## Introduction

The Wnt/β-catenin pathway, which is activated by the interaction of Wnt1, Wnt3a, and Wnt8 with Frizzled (Fz) receptors and low-density lipoprotein receptor-related protein5/6 (LRP5/6) co-receptors, plays important roles in cell proliferation, differentiation, and oncogenesis [1]. Central to this pathway is the level of cytosolic β-catenin, which regulates its target genes. In the absence of a Wnt signal, β-catenin is phosphorylated by both casein kinase 1 (CK1) and glycogen synthase kinase-3β (GSK-3β), which form a complex with adenomatous polyposis coli (APC)/Axin (destruction complex). This is then recognized by F-box β-transducin repeat-containing protein (β-TrCP), a component of the ubiquitin ligase complex, which results in the degradation of β-catenin [2]–[4]. Activation of the receptor by its Wnt ligands negatively regulates the destruction complex and leads to cytoplasmic β-catenin stabilization [5].

Abnormal activation of the Wnt/β-catenin pathway and subsequent up-regulation of β-catenin response transcription (CRT) is thought to contribute to the development and progression of certain cancers [6]. Oncogenic mutation in β-catenin or other components of the destruction complex (APC or Axin) are observed in colon cancer, hepatocelluar carcinoma, and prostate cancer [6]–[8]. These mutations lead to the excessive accumulation of β-catenin in cytoplasm and then β-catenin is translocated into the nucleus, where it complexes with T cell factor/lymphocyte enhancer factor (TCF/LEF) family transcription factors to activate the expression of Wnt/β-catenin responsive genes, such as *c-myc*, *cyclin D1* and metalloproteinase-7 (*MMP-7*), which play important roles in tumorigenesis and metastasis [9]–[11]. The accumulation of β-catenin is also observed in other types of cancer, such as ovarian cancer, melanoma, endometrial cancer, medulloblastoma, and pilomatricoma [6]–[8]. Thus, aberrant activation of the Wnt/β-catenin pathway is a potential therapeutic target for chemoprevention and treatment of various cancers.

In the present study, we identified CGK062, which markedly inhibits the Wnt/β-catenin pathway and cell proliferation of CRT-positive cancer cells. CGK062 promoted the degradation of intracellular β-catenin through PKCα-mediated β-catenin phosphorylation.

## Results

### Identification of CGK062 as an inhibitor of the Wnt/β-catenin pathway

**Figure 1:**
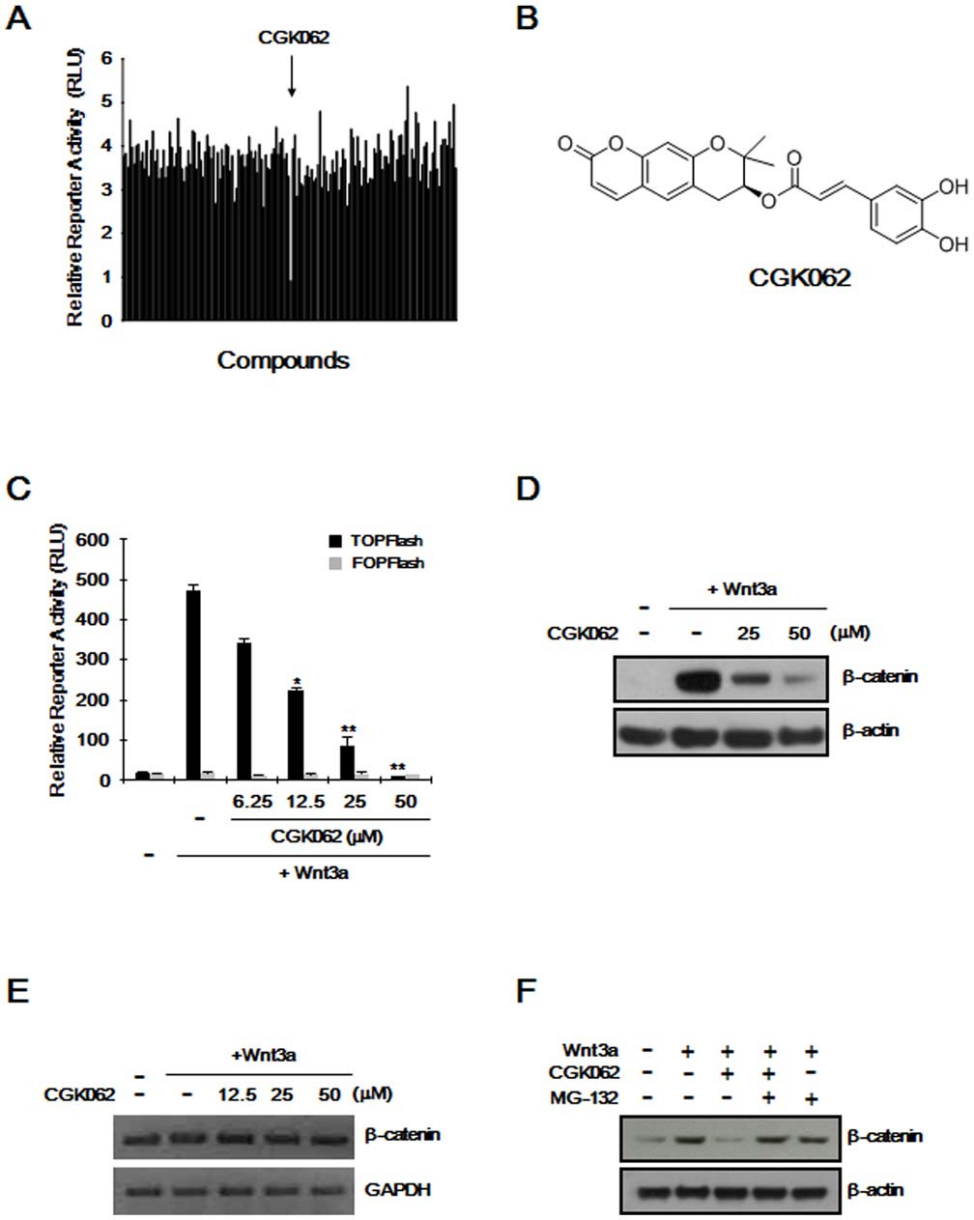
Identification of CGK062 as a potent small-molecule inhibitor of Wnt/β-catenin pathway. (A) Screening of compounds that inhibit the Wnt/β-catenin pathway. Compounds modulating TOPFlash reporter activity were screened using the HEK293 reporter cells. The controls were assayed in the presence or absence of Wnt3a-CM. (B) Chemical structure of CGK062. (C) Dose-dependent inhibition of β-catenin response transcription. HEK293 reporter cells were incubated with indicated concentrations of CGK062 in the presence of Wnt3a-CM. After 15 h, luciferase activities were determined and reported as relative light unit (RLU) normalized to cell titer. The results are the average of three experiments, and the bars indicate standard deviations. *, *P*<0.05, and **, *P*<0.01, compared with the Wnt3a-CM-treated control group. (D) Cytosolic proteins were prepared from HEK293 reporter cells treated with either vehicle (DMSO) or indicated concentrations of CGK062 in the presence of Wnt3a-CM for 15 h and then subjected to Western blotting with β-catenin antibody. (E) Semi-quantitative RT-PCR for β-catenin, and GAPDH was performed with total RNA prepared from HEK293 reporter cells either vehicle (DMSO) or indicated concentrations of CGK062 in the presence of Wnt3a-CM for 15 h. (F) Cytosolic proteins prepared from HEK293 reporter cells, which were incubated with vehicle (DMSO) or CGK062 (25 µM) in the presence or absence of Wnt3a-CM, exposed to MG-132 (10 µM) for 8 h, were subjected to Western blotting with anti-β-catenin antibody. In (D) amd (F), to confirm equal loading, the blot was reprobed with anti-actin antibody.

To identify small molecule antagonists of the Wnt/β-catenin pathway, we used HEK293 reporter cells, which stably harbored TOPFlash reporter and human Frizzled-1 (hFz-1) plasmids. After the incubation of these reporter cells with Wnt3a-CM and each compound, we measured firefly luciferase activity using a microplate reader and then identified that CGK062 (3-(3,4-dihydroxy-phenyl)-acrylic acid 2,2-dimethyl-8-oxo-3,4-dihydro-2H,8H-pyrano[3,2-g]chromen-3-yl ester) as an inhibitor of the Wnt/β-catenin pathway (Figure 1A, B and S1). As shown in Figure 1C, treatment of HEK293 reporter cells with different concentrations of CGK062 resulted in a dose-dependent decrease in β-catenin response transcription (CRT) that had been induced by Wnt3a-CM (IC_50_ = 12.3 µM). CGK062 did not affect FOPFlash activity in HEK293 control cells and cell viability in HEK293 reporter cells (Figure 1C and S2A). NF-κB and p53 reporter activities were not affected by CGK062 (Figure S2B and S2C). These results suggest that CGK062 potently and specifically inhibits the Wnt/β-catenin pathway.

In the Wnt/β-catenin pathway, CRT is largely dependent on the level of intracellular β-catenin, which is controlled by ubiquitin-dependent proteolysis [12]. To investigate the effect of CGK062 on the intracellular level of β-catenin, we analyzed the amount of cytoplasmic β-catenin by Western blotting with anti-β-catenin antibody in CGK062-treated HEK293 reporter cells. The level of cytoplasmic β-catenin, which had been accumulated by Wnt3a-CM, was dramatically decreased by treatment of CGK062 (Figure 1D), which is consistent with the CRT result. To examine whether the reduction of cytoplasmic β-catenin protein by this compound was due to the decrease of β-catenin mRNA level in HEK293 reporter cells, we performed semi-quantitative RT-PCR to determine the amount of β-catenin mRNA. As shown in Figure 1E, the β-catenin mRNA level did not change in response to different concentrations of CGK062 in HEK293 reporter cells.

Next, to explore whether down-regulation of β-catenin by CGK062 is mediated by the proteasome, we used MG-132 to inhibit proteasome-mediated protein degradation in HEK293 reporter cells. As shown in Figure 1F, CGK062 consistently led to a decrease in the β-catenin protein level; however, the addition of MG-132 abrogated the effect of CGK062 on the reduction in β-catenin. Ammonium chloride, a lysosome inhibitor, did not affect CGK062-mediated β-catenin degradation (Figure S3). Taken together, these results indicate that CGK062 suppresses the Wnt/β-catenin pathway via a mechanism involving the degradation of intracellular β-catenin.

### CGK062-mediated β-catenin degradation requires β-TrCP but not GSK-3βactivity

**Figure 2:**
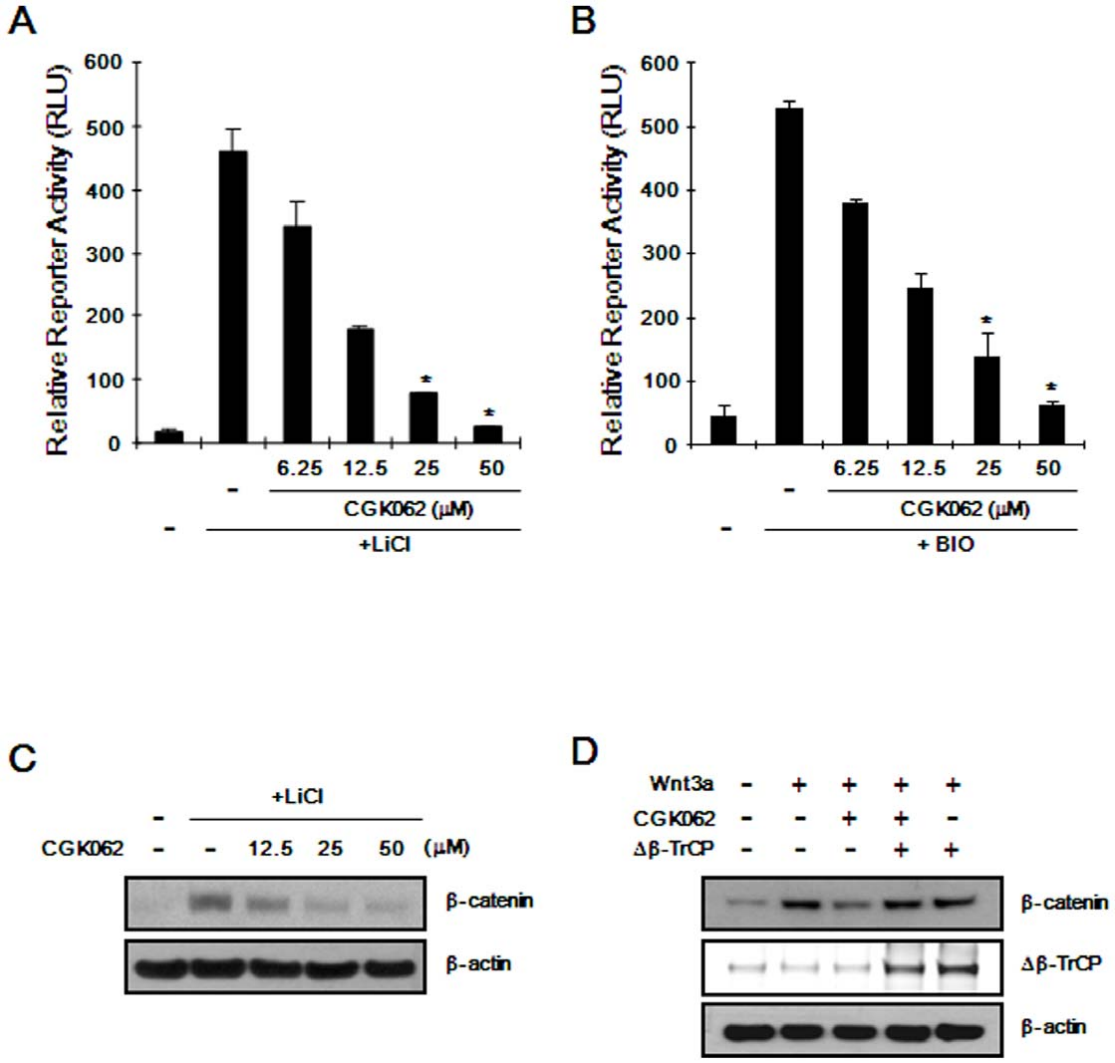
CGK062 induces β-catenin degradation through a mechanism independent of GSK-3β and dependent of β-TrCP. (A) HEK293 reporter cells were incubated with CGK062 in the presence of 20 mM LiCl. (B) HEK293 reporter cells were incubated with CGK062 in the presence of 0.75 µM BIO. In (A) and (B), after 15 h, luciferase activities were determined and reported as relative light unit (RLU) normalized to cell titer. The results are the average of three experiments, and the bars indicate standard deviations. *, *P*<0.05, compared with the LiCl- or BIO-treated control group. (C) Cytosolic proteins were prepared from HEK293 reporter cells treated with either vehicle (DMSO) or increasing amounts of CGK062 in the presence of 20 mM LiCl for 15 h and then subjected to Western blotting with β-catenin antibody. (D) HEK293 cells were transfected with the Δβ-TrCP expression plasmid and then incubated with either the vehicle (DMSO) or CGK062 (25 µM) in the presence of Wnt3a CM for 15 h. Cytosolic proteins were subjected to Western blotting with β-catenin and β-TrCP antibodies. In (C) and (D), the blots were reprobed with anti-actin antibody as a loading control.

Given that the phosphorylation of β-catenin by GSK-3β and its subsequent association with β-TrCP leads to β-catenin degradation [13], we examined whether GSK-3β activity is necessary for the degradation of β-catenin induced by CGK062. When HEK293 reporter cells were incubated with LiCl or 6-bromoindirubin-3′-oxim (BIO), inhibitors of GSK-3β, CRT was stimulated (Figure 2A and 2B), consistent with previous reports [14], [15]. As shown in Figure 2A and 2B, treatment with CGK062 resulted in the suppression of CRT in a dose-dependent manner. In addition, Western blot analysis using anti-β-catenin antibody consistently showed that CGK062 led to a down-regulation of intracellular β-catenin levels, which accumulated with LiCl (Figure 2C), suggesting that CGK062-mediated β-catenin degradation is independent of GSK-3β.

We then determined whether β-TrCP is necessary for CGK062-induced degradation ofβ-catenin. As shown in Figure 2D, ectopic expression of a dominant–negative form of β-TrCP (Δβ-TrCP), which interacts with phosphorylated β-catenin but is unable to form a SCF^β-TrCP^ ubiquitin ligase complex [16], abrogated the CGK062-induced degradation of β-catenin. In addition, CGK062 did not affect CRT that had been activated by overexpression of β-catenin mutants, S45A and S37A (Figure S4). These results indicate that β-TrCP and N-terminal residues of β-catenin are required for CGK062-mediated β-catenin degradation.

### CGK062 induces PKCα-mediated β-catenin phosphorylation/degradation

**Figure 3:**
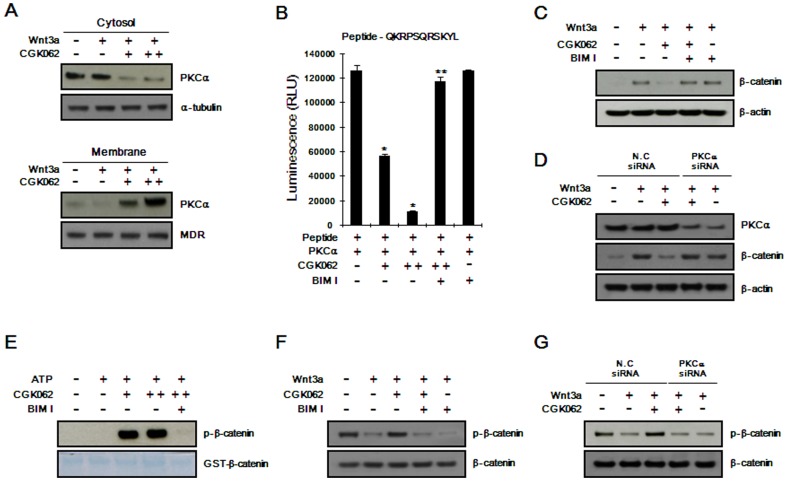
CGK062 promotes PKCα-mediated β-catenin phosphorylation/degradation. (A) Cytosolic and membrane fractions were prepared from HEK293 cells treated with vehicle (DMSO) or CGK062 (25 and 50 µM) in the presence or absence of Wnt3a-CM for Western blotting with anti-PKCα antibody. The blots were reprobed with anti-tubulin or anti-MDR antibodies as fraction controls. (B) Indicated peptide was incubated with purified PKCα and CGK062 (12.5 and 25 µM) or BIM (0.5 µM). The amounts of ATP in the reaction were detected by Kinase-Glo™ Luminescent Assay kit (Promega). The results are the average of three experiments, and the bars indicate standard deviations. *, *P*<0.05, compared with the vehicle control group. (C) Cytosolic proteins were prepared from HEK293 reporter cells treated with CGK062 (25 µM) or BIM (10 µM) in the absence or presence of Wnt3a-CM for Western blotting with anti-β-catenin antibody. (D) HEK293 reporter cells were transfected with negative control (NC) siRNA (40 nM) or PKCα siRNA (40 nM), and then incubated with CGK062 (25 µM) for 15 h in the absence or presence of Wnt3a-CM. Cell lysates were subjected to Western blot analysis with anti-PKCα and anti-β-catenin antibodies. In (C) and (D), the blots were reprobed with anti-actin antibody to confirm equal loading. (E) GST-β-catenin (100 ng) was incubated with purified PKCα and CGK062 (12.5 and 25 µM) or BIM (0.5 µM). The samples were analyzed by Western blotting with anti-phospho-p33/37-β-catenin antibody. (F) HEK293 reporter cells were incubated with CGK062 (25 µM) and BIM (10 µM) for 15 h in the absence or presence of Wnt3a-CM. Cytosolic fractions were prepared and subjected to Western blot analysis with anti-phospho-p33/37-β-catenin or anti-β-catenin antibody. (G) HEK293 reporter cells were transfected with negative control (NC) siRNA (40 nM) or PKCα siRNA (40 nM), and then incubated with CGK062 (25 µM) for 15 h in the absence or presence of Wnt3a-CM. Cell lysates were subjected to Western blot analysis with anti-β-catenin and anti-p33/37-β-catenin antibodies. In (F) and (G), the same amount of β-catenin was loaded in each lane.

Previous reports have demonstrated that activated PKCα catalyzes the phosphorylation of β-catenin at Ser33/37 and its subsequent association with β-TrCP leads to β-catenin degradation [17], [18]. To further gain insight into the mechanism, we first examined whether PKCα is activated by treatment with CGK062. Since activated PKCα moves from the cytoplasm to the plasma membrane [19], we isolated membrane fractions from CGK062-treated and -untreated cells, and measured the amount of PKCα by Western blot analysis. CGK062 treatment led to the accumulation of PKCα at the plasma membrane in HEK293 cells (Figure 3A). We also observed CGK062-induced membrane translocation of PKCα in HEK293 cells by immunofluorescence analysis (Figure S5). Consistently, CGK062 enhanced the kinase activity of PKCα *in vitro*, when we used synthetic peptide containing PKCα phosphorylation site as a substrate (Figure 3B). Moreover, BIM I, a specific inhibitor of PKC abolished the effect of CGK062 (Figure 3B), suggesting that CGK062 is a *bona fide* activator of PKCα.

We then examined whether PKCα activity is essential for CGK062-mediated β-catenin degradation. The inhibition of PKCα activity using BIM I abolished the down-regulation of β-catenin by CGK062 (Figure 3C). Notably, the selective depletion of endogenous PKCα using small-interfering RNA (siRNA) also nullified the CGK062-induced degradation ofβ-catenin (Figure 3D), indicating that PKCα is responsible for the degradation of β-catenin by CGK062.

Next, to test whether CGK062 directly promotes PKCα-mediated β-catenin phosphorylation at Ser33/37, we performed an *in vitro* kinase assay using bacterially expressed β-catenin and purified PKCα. PKCα readily phosphorylated β-catenin in the presence of CGK062 and BIM I inhibited this phosphorylation (Figure 3E). We also examined whether CGK062 promotes PKCα-mediated β-catenin phosphorylation at Ser33/37 and Ser45 in HEK293 reporter cells. Western blot analysis showed that Wnt3a-CM inhibited the phosphorylation of β-catenin at Ser33/37 and Ser45 (Figure 3F, S6 and S7). In addition, CGK062 induced the phosphorylation of β-catenin at Ser33/37 and Ser45 (Figure 3F, S6 and S7), and Ser33/37 phosphorylation was abrogated by adding BIM I (Figure 3F). Consistently, CGK062 treatment rescued the phosphorylation of β-catenin at Ser33/37, which was inhibited by Wnt3a-CM, and the knockdown of PKCα markedly suppressed CGK062-induced Ser33/37 phosphorylation in HEK293 reporter cells (Figure 3G).

### CGK062 also promotes β-catenin degradation in CRT-positive cancer cells

**Figure 4:**
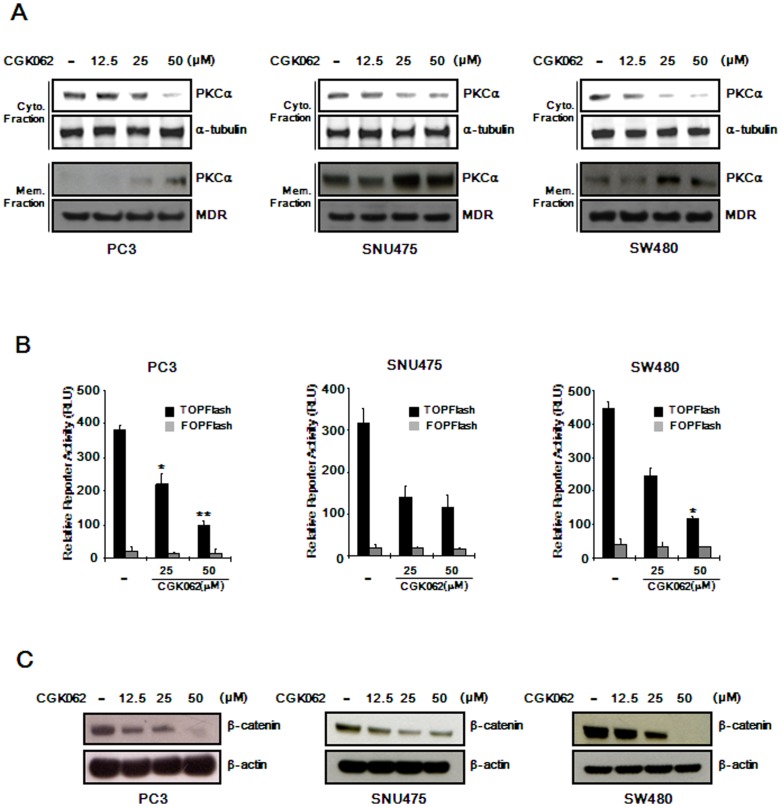
CGK062 promotes β-catenin degradation in CRT-positive cells. (A) Cytosolic and membrane fractions were prepared from PC3, SNU475, and SW480 cells treated with vehicle (DMSO) or CGK062 for Western blotting with anti-PKCα antibody. The blots were reprobed with anti-tubulin or anti-MDR antibodies as fraction controls. (B) PC3, SNU475, and SW480 cells were co-transfected with TOPFlash and pCMV-RL plasmids and incubated with CGK062 for 15 h. Luciferase activities were measured 39 h after transfection and reported as relative light unit (RLU) normalized to *Renilla* luciferase activities. Results are the average of three experiments, and the bars indicate standard deviations. *, *P*<0.05, and **, *P*<0.01, compared with the vehicle control group. (C) Cytosolic proteins were prepared from PC3, SNU475, and SW480 cells treated with the vehicle (DMSO) or CGK062 for 15 h and then subjected to Western blotting with β-catenin antibody. The blots were reprobed with anti-actin antibody to confirm equal loading.

We next tested whether CGK062 activates PKCα in CRT-positive cancer cells, such as PC3 (prostate cancer), SNU475 (hepatoma), and SW480 (colon cancer). Consistent with results from HEK293 cells, CGK062 promoted the translocation of PKCα to the plasma membrane in these cancer cells (Figure 4A). To determine whether CGK062 also inhibits β-catenin function in CRT-positive cancer cells, TOPFlash plasmid was transfected into CRT-positive cancer cells followed by treatment with increasing concentrations of CGK062. As shown in Figure 4B, CGK062 consistently repressed CRT in PC3, SNU475, and SW480 cells. In parallel with this experiment, we determined the effect of CGK062 on the level of cytosolic β-catenin in these CRT-positive cancer cells by Western blot analysis. Consistently, treatment of CGK062 resulted in the down-regulation of intracellular β-catenin level in a concentration-dependent manner in PC3, SNU475, and SW480 cells (Figure 4C). We also found that CGK062 promoted the phosphorylation of β-catenin at Ser33/37, and this phosphorylation was abolished by BIM I in SW480 cells (Figure S8). These results indicate that CGK062 also induces β-catenin degradation in CRT-positive cancer cells.

### CGK062 represses the expression of β-catenin-dependent genes

**Figure 5:**
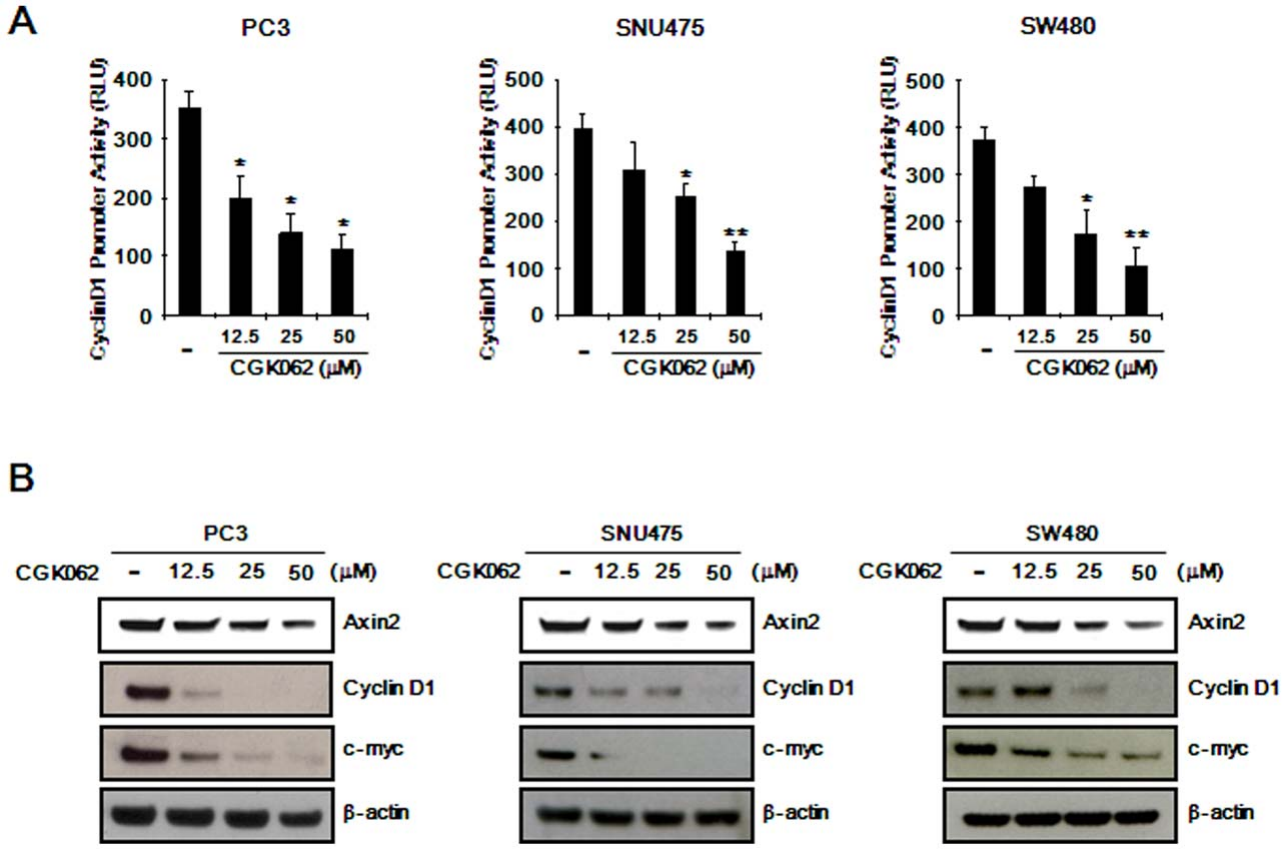
CGK062 represses the expression of the β-catenin-dependent genes. (A) PC3, SNU475, and SW480 cells were co-transfected with cyclin D1-RL and pSV40-FL, and then incubated with indicated amounts of CGK062 for 15 h. Luciferase activities were measured 39 h after transfection. Cyclin D1 promoter activity is reported as relative light unit (RLU) normalized to firefly luciferase activity. The results are the average of three experiments, and the bars indicate standard deviations. *, *P*<0.05, and **, *P*<0.01, compared with the vehicle control group. (B) PC3, SNU475, and SW480 cells were incubated with the vehicle (DMSO) or CGK062 for 15 h and then cell extracts were prepared for Western blotting with anti-axin, anti-cyclin D1 and anti-myc antibodies. To confirm equal loading, the blots were reprobed with anti-actin antibody.

To determine whether CGK062 affects the expression of β-catenin-dependent genes, the promoter activity of *cyclin D1*, which is a known β-catenin-dependent gene, was evaluated. A reporter construct containing the *cyclin D1* promoter, which contains a β-catenin/TCF-4 responsive region, was transfected into PC3, SNU475, and SW480 cells followed by treatment with different concentrations of CGK062. As shown in Figure 5A, *cyclin D1* promoter activity was repressed by CGK062 in these CRT-positive cancer cells. We also evaluated the protein level of cyclin D1 in CGK062-treated CRT-positive cancer cells. Consistent with our result for the *cyclin D1* promoter, a dose-dependent decrease in cyclin D1 protein expression was observed in response to CGK062 (Figure 5B) in PC3, SNU475, and SW480 cells. In addition, the expression of *c-myc* and *axin-2*, established downstream targets of β-catenin [9], were also significantly reduced in these CRT-positive cancer cells following incubation with CGK062 (Figure 5B). Under these conditions, the PKCα level did not change in response to different concentrations of CGK062 (Figure S9).

### CGK062 inhibits the proliferation of CRT-positive cancer cells *in vitro* and *in vivo*

Recent studies have demonstrated that the disruption of β-catenin function by antisense, siRNA, or secreted Wnt antagonist strategies has been shown to specifically inhibit the growth of CRT-positive cancer cells [20]–[22]. Given that CGK062 promoted the degradation of intracellular β-catenin, we hypothesized that CGK062 also suppresses the proliferation of CRT-positive cancer cells. To explore this hypothesis, we evaluated the effect of CGK062 on the growth of various CRT-positive cancer cells. As shown in Table 1 and Figure S10, CGK062 efficiently inhibited the growth of CRT-positive colon cancer cells (DLD-1, SW480, HCT15, SNU475, and PC3 cells) with IC_50_ values from 1.62 µM to 18.60 µM. Taken together, these results indicated that CGK062 markedly inhibits cell proliferation of CRT-positive cancer cells.

**Table 1:** IC_50_ values of CGK062.

Cell line	Type	IC_50_ (µM)
DLD-1	Colorectal Carcinoma	**18.60**
SW480	**Colorectal Carcinoma**	**1.62**
HCT15	**Colorectal Carcinoma**	**5.71**
SNU475	**Hepatocellular Carcinoma**	**11.41**
PC3	**Prostate Carcinoma**	**9.40**
WI38	**Normal Fibroblast**	**74.83**

**Figure 6:**
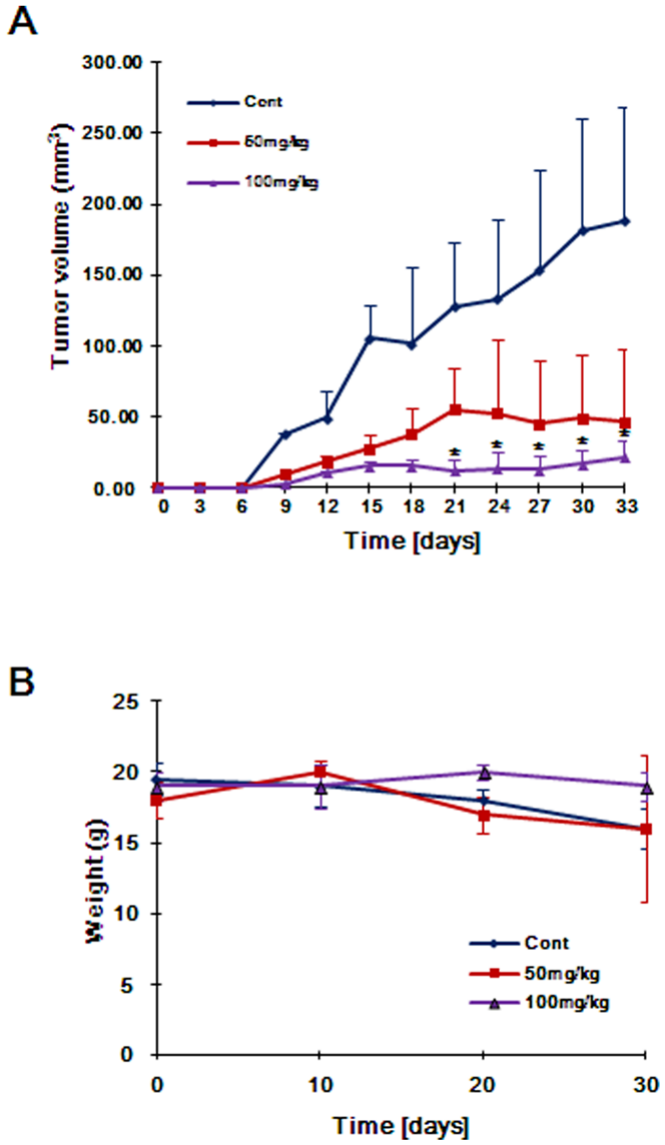
CGK062 inhibits the growth of CRT-positive cancer cells *in vivo*. (A and B) CGK062 inhibits prostate cancer tumor xenograft growth *in vivo*. Subcutaneous PC3 tumor xenografts were established and treatments were administered as described in Materials and Methods. (A), mean tumor volumes (n = 6) (B), mean average body weight changes during treatment. *, *P*<0.05, compared with the vehicle control group.

To further evaluate the antitumor activity of CGK062 *in vivo*, athymic nude mice bearing established s.c. PC3 xenograft tumors were treated daily with i.p. administration of CGK062 for 5 weeks at 50 and 100 mg/kg. In comparison to the vehicle (control), treatment of mice with CGK062 significantly inhibited PC3 tumor growth (Figure 6A). A dose of 100 mg/kg CGK062 induced near complete inhibition of tumor growth. Even at the dose of 50 mg/kg, CGK062 inhibited tumor growth by ∼70% relative to the control group. All mice tolerated the treatment without significant loss in body weight (Figure 6B).

## Discussion

Aberrant up-regulation of intracellular β-catenin is involved in the development of several cancers, including prostate cancer, colorectal cancer, and hepatocellular carcinoma [6]–[8]. In this report, we used a cell-based screening to identify CGK062 as a potent inhibitor of the Wnt/β-catenin pathway. CGK062 provided considerable therapeutic advantage with respect to antitumor potency in various β-catenin response transcription (CRT)-positive cancer cells, such as colon cancer cells (SW480, DLD-1, and HCT15), hepatocellular carcinoma (SNU475), hormone-refractory prostate cancer cells (PC3).

CGK062-activated PKCα catalyzed the phosphorylation of β-catenin at Ser33/Ser37, and thereby reduced the intracellular β-catenin level by β-TrCP-dependent proteasomal degradation. In addition, pharmacological inhibition and PKCα siRNA abrogated CGK062-induced β-catenin phosphorylation/degradation, indicating that PKCα is responsible for CGK062-mediated inhibition of the Wnt/β-catenin pathway. The results of several studies, including some conducted previously in our laboratory, suggest a key role for PKCα in the regulation of Wnt/β-catenin signaling. Wnt5a is involved in the mobilization of intracellular Ca^2+^, the subsequent activation of PKCα, and inhibition of the Wnt/β-catenin pathway [23], [24]. Orford *et al.* [25] reported that PKC inhibitors cause accumulation of β-catenin in human breast cancer cells. Recently, retinoic acid-related orphan nuclear receptor α (ROR α) was shown to attenuate Wnt/β-catenin signaling in colon cancer by stimulating PKCα-dependent phosphorylation [26]. Previously, we demonstrated that PKCα negatively regulates Wnt/β-catenin signaling in HEK293 cells, which have normal Wnt/β-catenin pathway function [17] and that PKCα regulates the intracellular β-catenin level in colon cancer cells [18]. The present study extends those previous findings by showing that the activation of PKCα by a novel agonist, CGK062, promotes β-catenin degradation in three cancer cell lines - PC3 (prostate cancer), SNU475 (hepatoma), and SW480 (colon cancer) -which display aberrant up-regulation of the intracellular β-catenin level.

Small molecule inhibitors that antagonize CRT have been discovered by high-throughput screening. Small molecules that block the association between Tcf4 and β-catenin impair β-catenin-dependent activities, such as cell proliferation and duplication of the embryonic dorsal axis in *Xenopus laevis* [27]. Another small molecule, ICG-001, which blocks the interaction between β-catenin and cyclic AMP response element-binding protein (CBP), specifically induces apoptosis in colon cancer cells [28]. Two other small molecules, IWR-3 and XAV939, are recently shown to stimulate the degradation of β-catenin by stabilizing axin and thereby inhibiting the proliferation of DLD-1 colon cancer cells, which carry a mutation in APC [29], [30]. In contrast to previously studied small molecules, CGK062 reduced the intracellular level of β-catenin through PKCα-mediated phosphorylation/degradation. Notably, it was able to promote β-catenin degradation in SNU475 hepatoma cells, which carry an axin mutation, as well as in SW480 colon cancer cells with APC mutation, and suppressed the growth of CRT-positive cancer cells. CGK062 markedly suppressed the growth of PC3 prostate tumors in a mouse xenograft model, consistent with the results of our *in vitro* analyses.

In conclusion, we identified CGK062 that promotes PKCα-mediated β-catenin phosphorylation and its degradation. CGK062 repressed the expression of its target genes, including those encoding cyclin D1, c-myc and axin-2, thereby suppressing tumor growth *in vitro* and *in vivo*. Taken together, CGK062 represents a promising candidate treatment of CRT-positive cancers.

## Materials and Methods

### Cell culture, plasmids, transfection, and luciferase assay

HEK293, PC-3, SNU475, DLD-1, HCT15, SW480, and Wnt3a-secreting L cells were obtained from American Type Culture Collection (ATCC) and maintained in Dulbecco's modified Eagle's medium (DMEM) supplemented with 10% fetal bovine serum (FBS), 120 µg/ml penicillin, and 200 µg/ml streptomycin. Wnt3a-conditioned medium (Wnt3a-CM) was prepared as previously described [31]. The HEK293 reporter and control cell line was established as previously described [32]. The pTOPFlash and pFOPFlash reporter plasmids were obtained from Upstate Biotechnology (Lake Placid, NY, USA). The dominant negative β-TrCP (Δβ-TrCP) expression plasmid was a gift from M. Davis (Hebrew University-Hadassah Medical School, Israel). pCMV-RL and pSV-FL plasmids were purchased from Promega. PKC siRNA was designed as previously described [17]. Transfection was performed with Lipofectamine 2000 (Invitrogen, Carlsbad, CA, USA) according to the manufacturer's instructions. The luciferase assay was performed using a Dual Luciferase Assay kit (Promega, Madison, WI, USA).

### Screening for a small-molecule inhibitor of Wnt/β-catenin signaling

The HEK293 reporter cells were inoculated into 96-well plates at 15,000 cells per well in duplicate and grown for 24 h. Wnt3a-CM was added, and then compounds (800 compounds) including coumarins, flavonoids, naphthoquinones, and terpenoids were added to the wells at a final concentration of 30 µM. After 15 h, the plates were assayed for firefly luciferase activity and cell viability.

### Preparation of the membrane fraction

Cells grown in 100-mm culture dishes were washed with ice-cold PBS. The cells were then suspended in 1 ml of ice-cold extraction buffer (20 mM Tris (pH 7.5), 0.5 mM EDTA, and 0.5 mM EGTA); homogenized using a syringe (26G); and incubated on ice for 30 min. The homogenate was centrifuged at 13,400× *g* for 2 min at 4°C. The supernatant was centrifuged at 100,000× *g* for 30 min at 4°C in a 100Ti rotor (Beckman, USA). The pellet was suspended in extraction buffer containing 0.5% (w/v) Triton X-100.

### Western blotting

The cytosolic fraction was prepared as previously described [33]. Proteins were separated by SDS-PAGE in a 4–12% gradient gel (Invitrogen, Carlsbad, CA, USA) and transferred to nitrocellulose membranes (Bio-Rad, Hercules, CA, USA). The membranes were blocked with 5% nonfat milk and probed with anti-β-catenin (BD Transduction Laboratories, Lexington, KY, USA), anti-phospho-β-catenin (Ser33/Ser37/Thr41) (Cell Signaling Technology, Beverly, MA, USA), anti-phospho-β-catenin (Ser45) (Cell Signaling Technology), anti-cyclin D1 (Santa Cruz Biotechnology, Santa Cruz, CA, USA), anti-myc (Santa Cruz Biotechnology), anti-PKCα (BD Transduction Laboratories), anti-axin (BD Transduction Laboratories), anti-β-TrCP (Santa Cruz Biotechnology), anti-MDR1 (Santa Cruz Biotechnology) and anti-actin antibodies (Cell Signaling Technology, Beverly, MA, USA). The membranes were then incubated with horseradish-peroxidase-conjugated anti-mouse IgG or anti-rabbit IgG (Santa Cruz Biotechnology) and visualized using the ECL system (Santa Cruz Biotechnology).

### RNA extraction and semi-quantitative RT-PCR

Total RNA was isolated with Trizol reagent (Invitrogen, Carlsbad, CA, USA) in accordance with the manufacturer's instructions. cDNA synthesis, reverse transcription, and PCR (Polymerase chain reaction) were performed as previously described [32]. The amplified DNA was separated on 2% agarose gels and stained with ethidium bromide.

### Immunofluorescence analysis

HEK293cells were cultured on glass chamber slides and then treated with DMSO or CGK062 for 15 h. After treatment, the cells were washed with PBS, fixed with 4% formaldehyde, permeabilized in 0.3% Triton X-100, and blocked in 4% bovine serum albumin for 1 h. The cells were stained with anti-PKCα or anti-E-cadherin (BD Transduction Laboratories) antibodies and then analyzed by confocal microscopy using a Zeiss LSM510 Meta microscope.

### In vitro kinase assay

Kinase assays were performed with purified PKCα (Sigma) using GST-β-catenin (100 ng) as a substrate as previously described [17]. The proteins were subjected to SDS-PAGE and transferred onto nitrocellulose membranes. The transferred proteins were analyzed using Western blotting with anti-phospho-β-catenin antibody (Cell Signaling Technology), and visualized using the ECL system (Santa Cruz Biotechnology).

### Cell viability assay

Cells were inoculated into 96-well plates and treated with CGK062 for 48 h. The cell viability from each treated sample was measured in triplicate using CellTiter-Glo assay kit (Promega, Madison, WI, USA) according to the manufacturer's instructions.

### In vivo xenograft experiment

Animal experiment was performed with approval of the Animal Study Committee of Inje University (Permit Number: 2008-051). PC3 prostate cancer cells (1×10^7^) cells were injected s.c. into 6-week-old female athymic nude mice. One week after cell implantation, mice were randomly assigned to three experimental groups (n = 5 each) and then treated with CGK062 (50 mg/kg/day or 100 mg/kg/day) by i.p. for 4 weeks. Tumors were measured with a caliper and their volumes were calculated using formula, width^2^×length×0.52.

## Supporting Information

Figure S1 **Schematic diagram of CGK062 synthesis.** CGK062 was synthesized by the following procedure. (+)-Decursinol was prepared form the roots of *A. Gigas* and was added to a solution of caffeic acid (5 g, 1eq) in pyridine (11.2 ml, 5eq) was added acetic anhydride (26.2 ml, 10eq) at room temperature. The reaction mixture was stirred for 1 day and extracted with ethylacetate/H_2_O (1∶1) solution. The organic layer was dried over anhydrous sodium sulfate, and concentrated in vacuo. The crude mixture was solidified with ethylacetate/*n*-hexane (1∶1) solution to give 3-(3,4-diacetoxy-phenyl)-acrylic acid in 74.8% yield (5.48 g). Thionyl chloride (2.37 ml, 5eq) was added to a solution of 3-(3,4-diacetoxy-phenyl)-acrylic acid (1.6 g, 1eq) in anhydrous benzene (18 ml) and catalytic amount of DMF (1 drop). The reaction mixture was refluxed for 6 h and then allowed to cooling to room temperature. After removal of solvent by rotary evaporation, the residue (intermediate A) was used next reaction without further purification. To a solution of decursinol (1.19 g, 0.8eq) and pyridine (1.17 ml, 2.4eq) in 30 ml of anhydrous methylene chloride was added a solution of intermediate A in 20 ml of anhydrous methylene chloride. After stirring for 5 h at room temperature, the solvent was removed in vacuo to give intermediate B, which was purified by a silica gel column chromatography in 84.5% yield (2.08 g). White Solid, mp: 92°C, R*_f_* = 0.27 (1∶1 *n*-hexane-ethyl acetate); [α]25 D+28.0 (c = 3, CHCl_3_); ^1^H NMR(400 MHz, CDCl_3_): δ_H_ 7.60(1H, d, *J* = 16.0 Hz, H-3'), 7.59(1H, d, *J* = 9.6 Hz, H-4), 7.38-7.33(2H, m, H-5', H-9'), 7.25-7.17(2H, m, H-5, H-8'), 6.82(1H, s, H-10), 6.35(1H, d, *J* = 16.0 Hz, H-2'), 6.23(1H, d, *J* = 9.2 Hz. H-3), 5.19(1H, t, *J* = 4.6 Hz, H-7), 3.24(1H, dd, *J* = 4.6, 17.6 Hz, H-6a), 2.93(1H, dd, *J* = 4.6, 17.6 Hz, H-6b), 2.29(3H, s, OAc-6'), 2.29(3H, s, OAc-7'), 1.42(3H, s, CH_3_-8), 1.38(3H, s, CH_3_-8); ^13^C NMR (100 MHz, acetone-d_6_) δ_C_ 168.5(OC = O-6'), 168.4(OC = O-7'), 166.2(C-1'), 160.8(C-2), 157.1(C-9a), 155.0(C-10a), 145.0(C-3'), 144.3(C-6'), 144.3(C-7'), 143.7(C-4), 133.7(C-4'), 130.2(C-5), 127.4(C-9'), 124.9(C-8'), 123.9(C-5'), 119.4(C-2'), 116.7(C-5a), 113.8(C-3), 113.7(C-4a), 104.6(C-10), 77.5(C-8), 70.9(C-7), 28.2(C-6), 25.0(CH_3_-8), 23.6(CH_3_-8), 20.4(OCOCH_3_-6'), 20.4(OCOCH_3_-7'); ESI-MS: m/z = 493 [M+H]^+^. Anal. Calc. for C_27_H_24_O_9_: C, 65.95; H, 4.91. Found: C, 65.88; H,4.90. To a solution of intermediate B (1.7 g, 1eq) in 60 ml of acetone was added 3N HCl (3 ml) and the reaction mixture was refluxed for 18 h. After cooling to room temperature, the reaction mixture was evaporated, extracted with ethylacetate/H_2_O (1∶1) and washed with brine. The washed solution was dried over anhydrous sodium sulfate, and concentrated in vacuo. The desired compound (CGK062) was obtained by short silica gel column chromatography in 93.2% yield (1.31 g). White Solid, mp: 115°C, R*_f_* = 0.36 (1∶2 *n*-hexane-ethyl acetate); [α]25 D+19.3 (c = 3, CHCl_3_); ^1^H NMR(400 MHz, DMSO-d_6_): δ_H_ 9.63(1H, s, OH-7'), 9.10(1H, s, OH-6'), 7.90(1H, d, *J* = 9.6 Hz, H-4), 7.46(1H, s, H-5), 7.45(1H, d, *J* = 15.2 Hz, H-3'), 7.00(1H, s, H-5'), 6.99(1H, d, *J* = 8.4 Hz, H-9'), 6.81(1H, s, H-10), 6.71(1H, d, *J* = 8.4 Hz, H-8'), 6.25(1H, d, *J* = 9.6 Hz, H-3), 6.22(1H, d, *J* = 15.6 Hz, H-2'), 5.14(1H, t, *J* = 4.0 Hz, H-7), 3.24(1H, dd, *J* = 4.0, 17.6 Hz, H-6a), 2.88(1H, dd, *J* = 4.0, 17.6 Hz, H-6b), 1.35(3H, s, CH_3_-8), 1.31(3H, s, CH_3_-8); ^13^C NMR (100 MHz, CDCl_3_): δ_C_ 166.9(C-1'), 162.3(C-2), 156.6(C-9a), 154.0(C-10a), 147.0(C-7'), 146.2(C-6'), 144.2(C-3'), 143.9(C-4), 128.8(C-5), 126.8(C-4'), 122.5(C-9'), 116.0(C-5a), 115.3(C-8'), 114.3(C-2'), 114.1(C-3), 112.8(C-4a), 112.7(C-5'), 104.7(C-10), 76.8(C-8), 70.0(C-7), 27.8(C-6), 24.8(CH_3_-8), 23.3(CH_3_-8); ESI-MS: m/z = 409 [M+H]^+^. Anal. Calc. for C_23_H_20_O_7_: C, 67.64; H, 4.94. Found: C, 67.58; H, 4.95.
(TIF)

Figure S2 **CGK062 is not cytotoxic to HEK293 reporter cells and does not affect other pathways.** (A) HEK293 reporter cells were incubated with CGK062 (12.5, 25 and 50 µM) for 15 h in the absence or presence of Wnt3a-CM, and cell viability was measured by Cell titer-Glo (Promega). (B) HCT116 cells were co-transfected with p53-FL and pCMV-RL plasmids and incubated with CGK062 in the presence or absence of doxorubicin, an activator of p53 pathway, for 15 h. Luciferase activities were measured 39 h after transfection and reported as relative light unit (RLU) normalized to *Renilla* luciferase activities. (C) HEK293 cells were co-transfected with NF-κB-FL and pCMV-RL plasmids and incubated with CGK062 in the presence or absence of aspirin, an activator of NF-κB pathway, for 15 h. Luciferase activities were measured 39 h after transfection and reported as relative light unit (RLU) normalized to *Renilla* luciferase activities.
(TIF)

Figure S3 **CGK062 induces β-catenin degradation through a mechanism independent of the lysosomal degradation pathway.** Cytosolic proteins prepared from HEK293 reporter cells, which were incubated with vehicle (DMSO) or CGK062 (25 µM) in the presence or absence of Wnt3a CM, exposed to NH_4_C1 (10 mM), were subjected to Western blotting with anti-β-catenin antibody.
(TIF)

Figure S4 **The N-terminus of β-catenin is required for CGK062-mediated β-catenin degradation.** HEK293 reporter cells were transfected with wild-type β-catenin (A), β-catenin S45A (B) or β-catenin S37A (C) plasmids, incubated with CGK062 for 15 h, and then luciferase activities were measured.
(TIF)

Figure S5 **CGK062 induces the translocation of PKCα to the membrane.** The cellular location of PKCα in HEK293 cells was determined by immunofluorescence analysis. HEK293 reporter cells were incubated with vehicle (DMSO) or CGK062 (25 µM) for 15 h. After fixation, the cells were stained with anti-E-cadherin and anti-PKCα antibody and observed at 400× magnification.
(TIF)

Figure S6 **CGK062 phosphorylates Ser45 residues of β-catenin.** HEK293 reporter cells were incubated with CGK062 for 15 h in the absence or presence of Wnt3a-CM. Cytosolic fractions were prepared and subjected to western blot analysis with anti-phospho-p45-β-catenin or anti-β-catenin antibody. The same amount of β-catenin was loaded in each lane.
(TIF)

Figure S7 **CGK062 promotes β-catenin phosphorylation and degradation.** Cytosolic proteins prepared from HEK293 reporter cells, which were incubated with vehicle (DMSO) or CGK062 in the presence of Wnt3a-CM for indicated periods of time, were subjected to Western blotting with anti-β-catenin antibody (A) or anti-phospho-p33/37/41-β-catenin antibody (B). In (B), the same amount of β-catenin was loaded in each lane.
(TIF)

Figure S8 **CGK062 promotes PKCα-mediated β-catenin degradation in SW480 cells.** SW480 cells were incubated with CGK062 (25 µM) and BIM (10 µM) for 15 h. Cytosolic fractions were prepared and subjected to Western blot analysis with anti-phospho-p33/37-β-catenin or anti-β-catenin antibodies. The same amount of β-catenin was loaded in each lane.
(TIF)

Figure S9 **CGK062 does not affect the expression of PKCα.** PC3 (A), SNU475 (B), and SW480 (C) cells were incubated with the vehicle (DMSO) or CGK062 for 15 h and then cell extracts were prepared for Western blotting with anti-PKCα antibody. To confirm equal loading, the blots were reprobed with anti-actin antibody.
(TIF)

Figure S10 **CGK062 inhibits the proliferation of CRT-positive cells.** Cells were incubated, in the indicated concentrations of CGK062, for 48 hrs in 96-well plates. Cell viability was examined using the CellTiter-Glo assay (Promega). To calculate the inhibition of growth, the value at time 0 was subtracted.
(TIF)
