# Supplementary figures and images for: Small Molecule-Based Promotion of PKCα-Mediated β-Catenin Degradation Suppresses the Proliferation of CRT-Positive Cancer Cells
Source: PLoS One. 2012 Oct 5;7(10):e46697. doi: 10.1371/journal.pone.0046697 (PMC3465275; doi:10.1371/journal.pone.0046697)

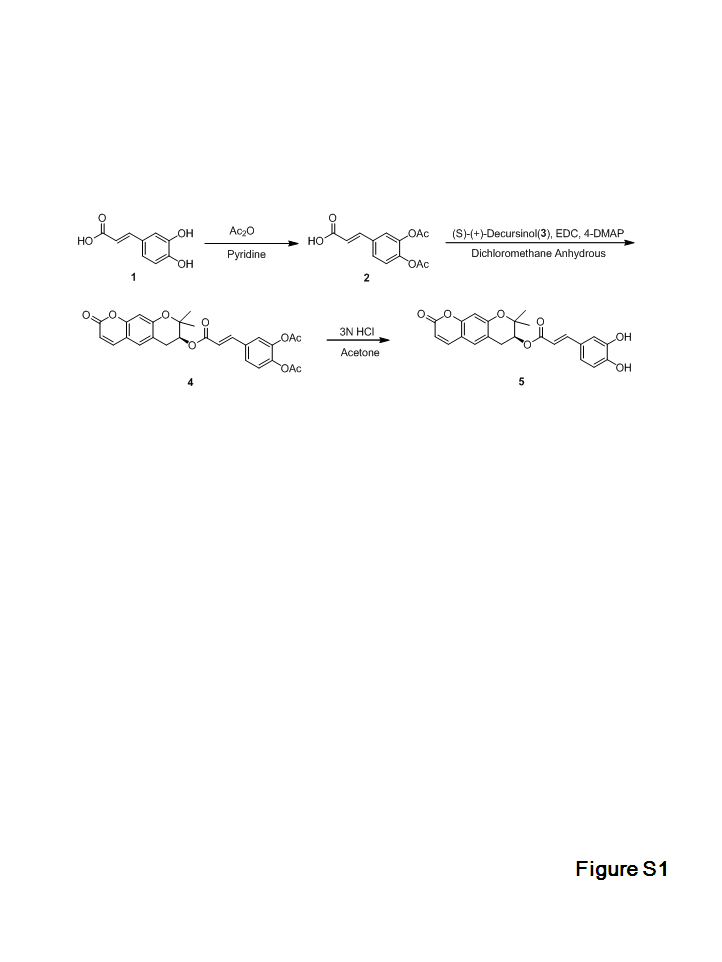

Supplement: Figure S1 — Schematic diagram of CGK062 synthesis. CGK062 was synthesized by the following procedure. (+)-Decursinol was prepared form the roots of A. Gigas and was added to a solution of caffeic acid (5 g, 1eq) in pyridine (11.2 ml, 5eq) was added acetic anhydride (26.2 ml, 10eq) at room temperature. The reaction mixture was stirred for 1 day and extracted with ethylacetate/H2O (1∶1) solution. The organic layer was dried over anhydrous sodium sulfate, and concentrated in vacuo. The crude mixture was solidified with ethylacetate/n-hexane (1∶1) solution to give 3-(3,4-diacetoxy-phenyl)-acrylic acid in 74.8% yield (5.48 g). Thionyl chloride (2.37 ml, 5eq) was added to a solution of 3-(3,4-diacetoxy-phenyl)-acrylic acid (1.6 g, 1eq) in anhydrous benzene (18 ml) and catalytic amount of DMF (1 drop). The reaction mixture was refluxed for 6 h and then allowed to cooling to room temperature. After removal of solvent by rotary evaporation, the residue (intermediate A) was used next reaction without further purification. To a solution of decursinol (1.19 g, 0.8eq) and pyridine (1.17 ml, 2.4eq) in 30 ml of anhydrous methylene chloride was added a solution of intermediate A in 20 ml of anhydrous methylene chloride. After stirring for 5 h at room temperature, the solvent was removed in vacuo to give intermediate B, which was purified by a silica gel column chromatography in 84.5% yield (2.08 g). White Solid, mp: 92°C, Rf = 0.27 (1∶1 n-hexane-ethyl acetate); [α]25 D+28.0 (c = 3, CHCl3); 1H NMR(400 MHz, CDCl3): δH 7.60(1H, d, J = 16.0 Hz, H-3'), 7.59(1H, d, J = 9.6 Hz, H-4), 7.38-7.33(2H, m, H-5', H-9'), 7.25-7.17(2H, m, H-5, H-8'), 6.82(1H, s, H-10), 6.35(1H, d, J = 16.0 Hz, H-2'), 6.23(1H, d, J = 9.2 Hz. H-3), 5.19(1H, t, J = 4.6 Hz, H-7), 3.24(1H, dd, J = 4.6, 17.6 Hz, H-6a), 2.93(1H, dd, J = 4.6, 17.6 Hz, H-6b), 2.29(3H, s, OAc-6'), 2.29(3H, s, OAc-7'), 1.42(3H, s, CH3-8), 1.38(3H, s, CH3-8); 13C NMR (100 MHz, acetone-d6) δC 168.5(OC = O-6'), 168.4(OC = O-7'), 166.2(C-1'), 160.8(C-2), 15 [file pone.0046697.s001.tif]

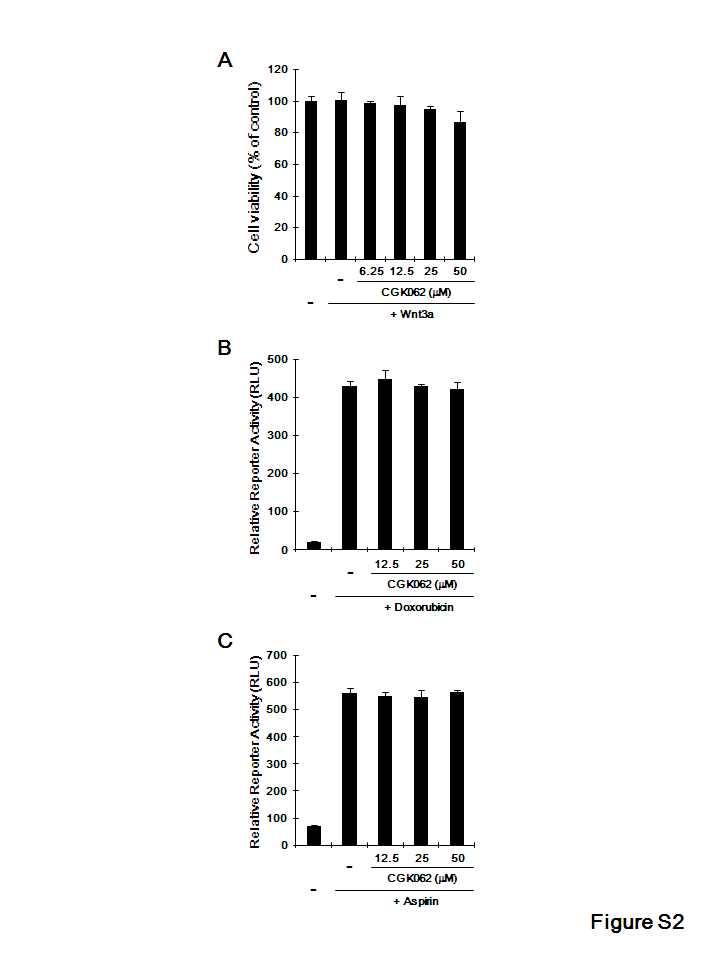

Supplement: Figure S2 — CGK062 is not cytotoxic to HEK293 reporter cells and does not affect other pathways. (A) HEK293 reporter cells were incubated with CGK062 (12.5, 25 and 50 µM) for 15 h in the absence or presence of Wnt3a-CM, and cell viability was measured by Cell titer-Glo (Promega). (B) HCT116 cells were co-transfected with p53-FL and pCMV-RL plasmids and incubated with CGK062 in the presence or absence of doxorubicin, an activator of p53 pathway, for 15 h. Luciferase activities were measured 39 h after transfection and reported as relative light unit (RLU) normalized to Renilla luciferase activities. (C) HEK293 cells were co-transfected with NF-κB-FL and pCMV-RL plasmids and incubated with CGK062 in the presence or absence of aspirin, an activator of NF-κB pathway, for 15 h. Luciferase activities were measured 39 h after transfection and reported as relative light unit (RLU) normalized to Renilla luciferase activities. (TIF) [file pone.0046697.s002.tif]

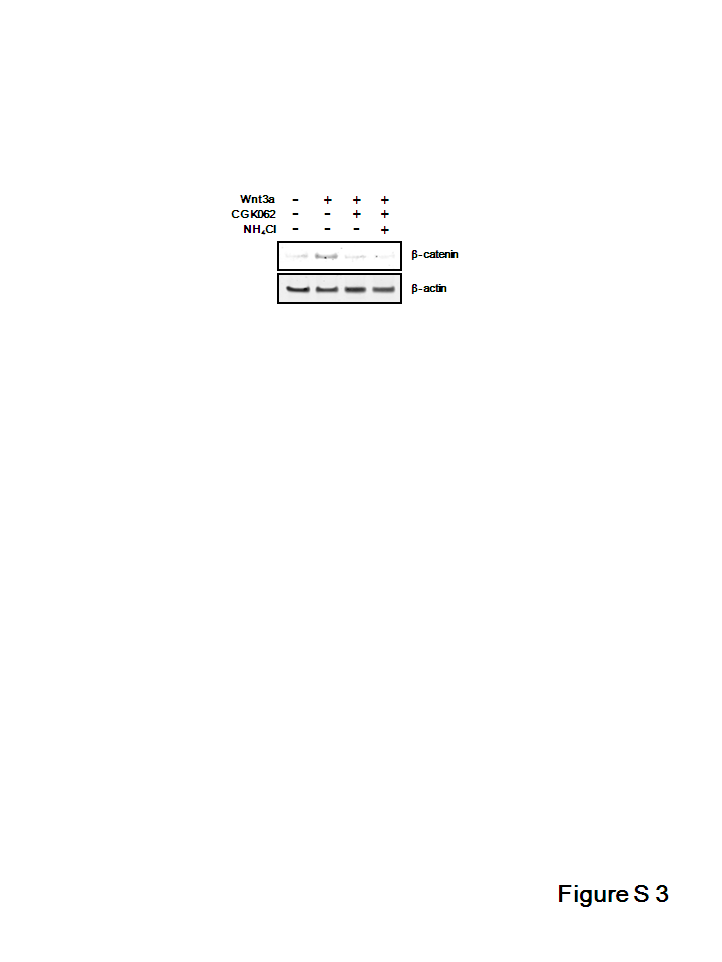

Supplement: Figure S3 — CGK062 induces β-catenin degradation through a mechanism independent of the lysosomal degradation pathway. Cytosolic proteins prepared from HEK293 reporter cells, which were incubated with vehicle (DMSO) or CGK062 (25 µM) in the presence or absence of Wnt3a CM, exposed to NH4C1 (10 mM), were subjected to Western blotting with anti-β-catenin antibody. (TIF) [file pone.0046697.s003.tif]

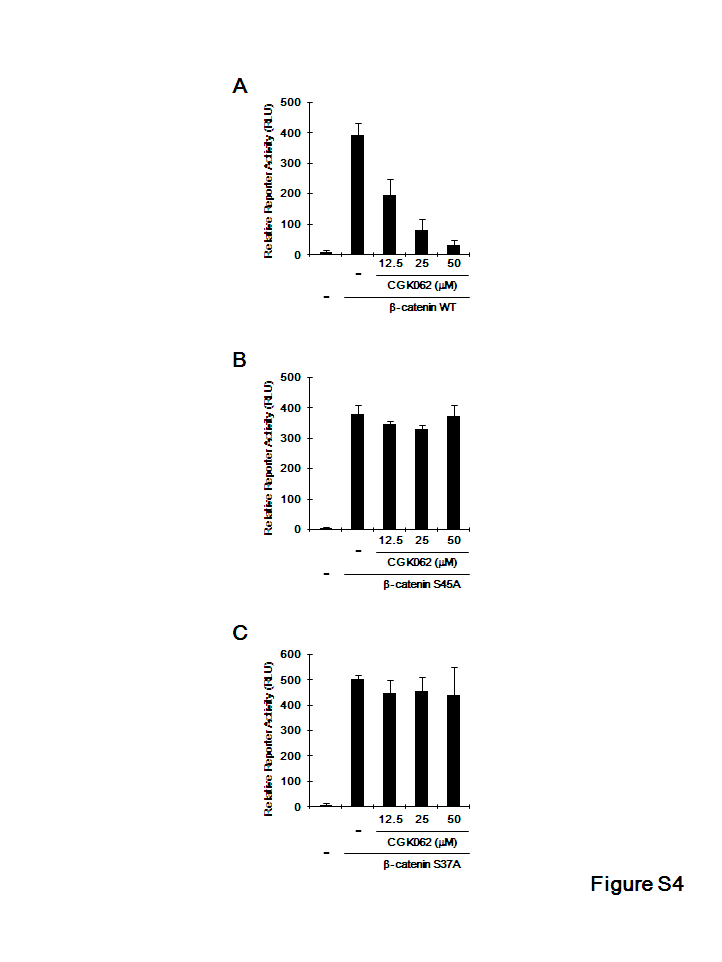

Supplement: Figure S4 — The N-terminus of β-catenin is required for CGK062-mediated β-catenin degradation. HEK293 reporter cells were transfected with wild-type β-catenin (A), β-catenin S45A (B) or β-catenin S37A (C) plasmids, incubated with CGK062 for 15 h, and then luciferase activities were measured. (TIF) [file pone.0046697.s004.tif]

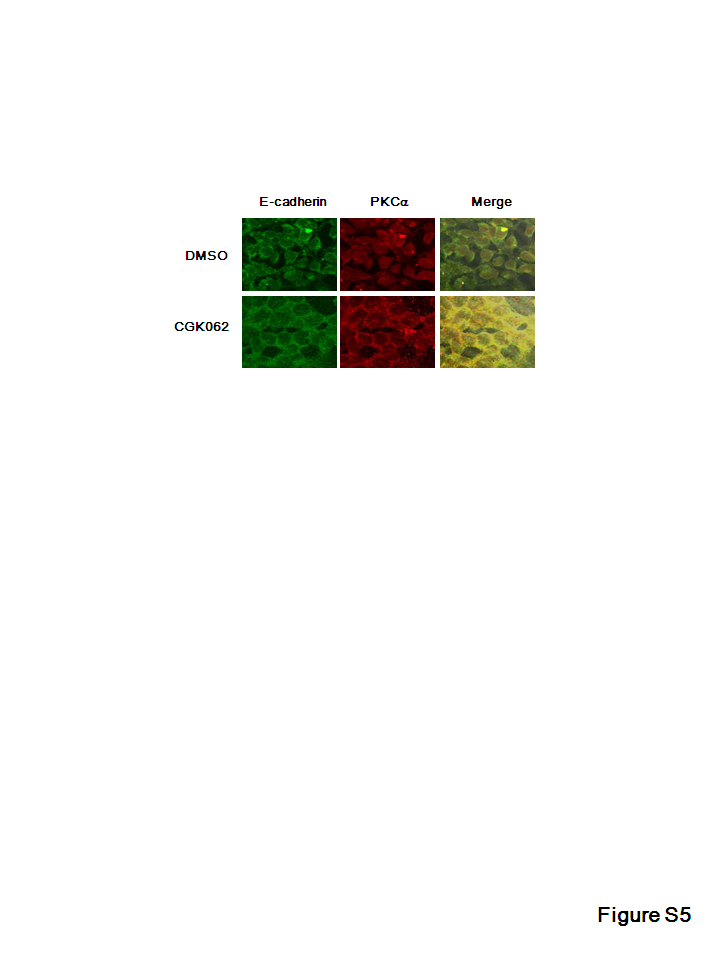

Supplement: Figure S5 — CGK062 induces the translocation of PKCα to the membrane. The cellular location of PKCα in HEK293 cells was determined by immunofluorescence analysis. HEK293 reporter cells were incubated with vehicle (DMSO) or CGK062 (25 µM) for 15 h. After fixation, the cells were stained with anti-E-cadherin and anti-PKCα antibody and observed at 400× magnification. (TIF) [file pone.0046697.s005.tif]

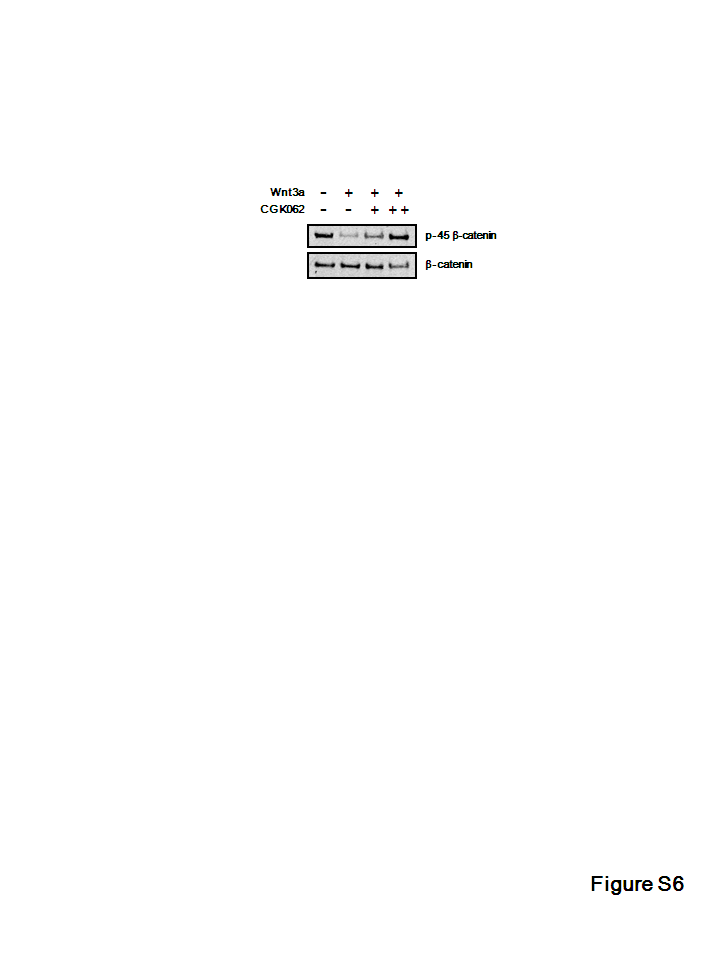

Supplement: Figure S6 — CGK062 phosphorylates Ser45 residues of β-catenin. HEK293 reporter cells were incubated with CGK062 for 15 h in the absence or presence of Wnt3a-CM. Cytosolic fractions were prepared and subjected to western blot analysis with anti-phospho-p45-β-catenin or anti-β-catenin antibody. The same amount of β-catenin was loaded in each lane. (TIF) [file pone.0046697.s006.tif]

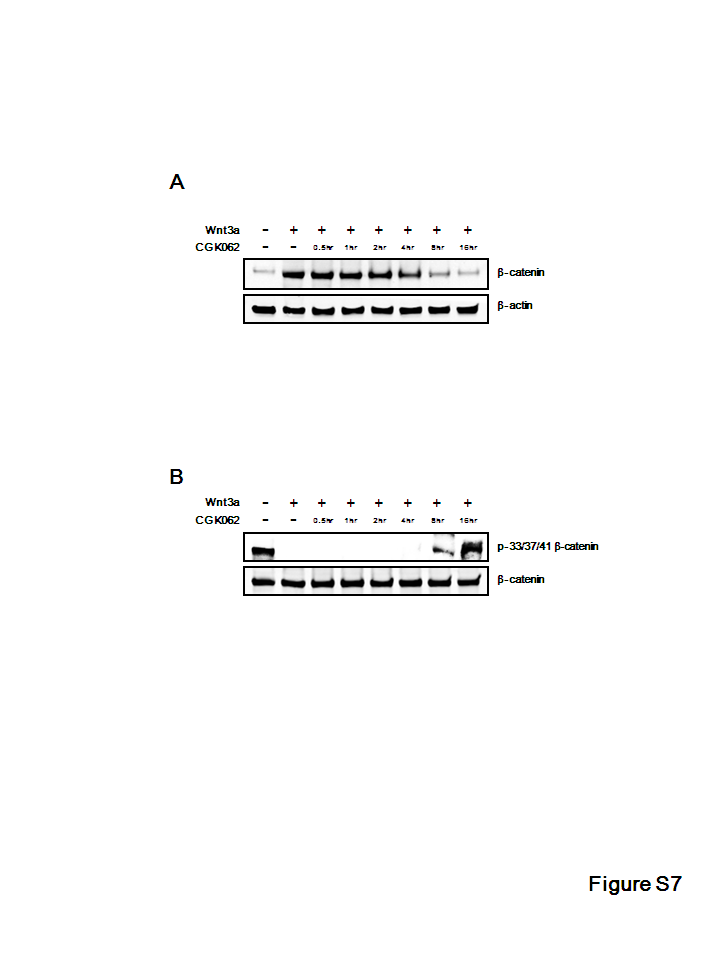

Supplement: Figure S7 — CGK062 promotes β-catenin phosphorylation and degradation. Cytosolic proteins prepared from HEK293 reporter cells, which were incubated with vehicle (DMSO) or CGK062 in the presence of Wnt3a-CM for indicated periods of time, were subjected to Western blotting with anti-β-catenin antibody (A) or anti-phospho-p33/37/41-β-catenin antibody (B). In (B), the same amount of β-catenin was loaded in each lane. (TIF) [file pone.0046697.s007.tif]

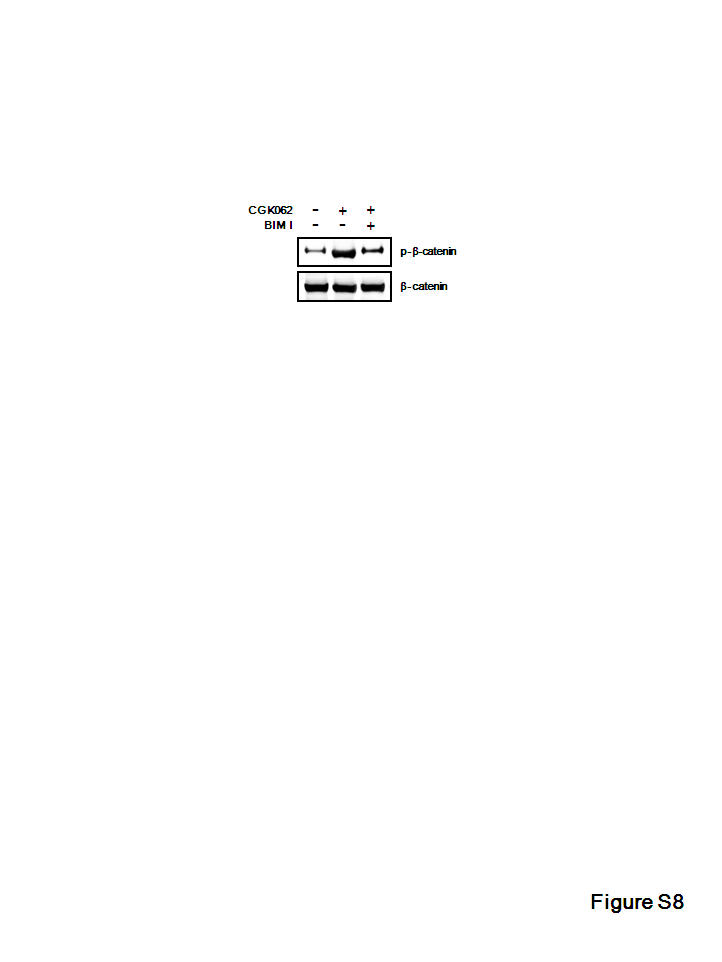

Supplement: Figure S8 — CGK062 promotes PKCα-mediated β-catenin degradation in SW480 cells. SW480 cells were incubated with CGK062 (25 µM) and BIM (10 µM) for 15 h. Cytosolic fractions were prepared and subjected to Western blot analysis with anti-phospho-p33/37-β-catenin or anti-β-catenin antibodies. The same amount of β-catenin was loaded in each lane. (TIF) [file pone.0046697.s008.tif]

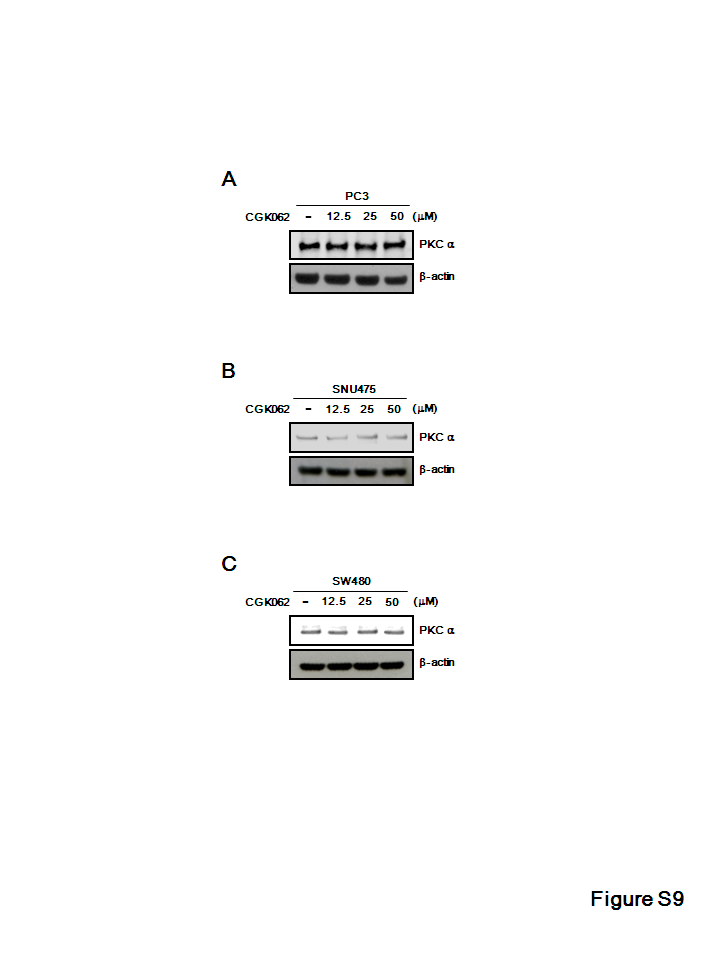

Supplement: Figure S9 — CGK062 does not affect the expression of PKCα. PC3 (A), SNU475 (B), and SW480 (C) cells were incubated with the vehicle (DMSO) or CGK062 for 15 h and then cell extracts were prepared for Western blotting with anti-PKCα antibody. To confirm equal loading, the blots were reprobed with anti-actin antibody. (TIF) [file pone.0046697.s009.tif]

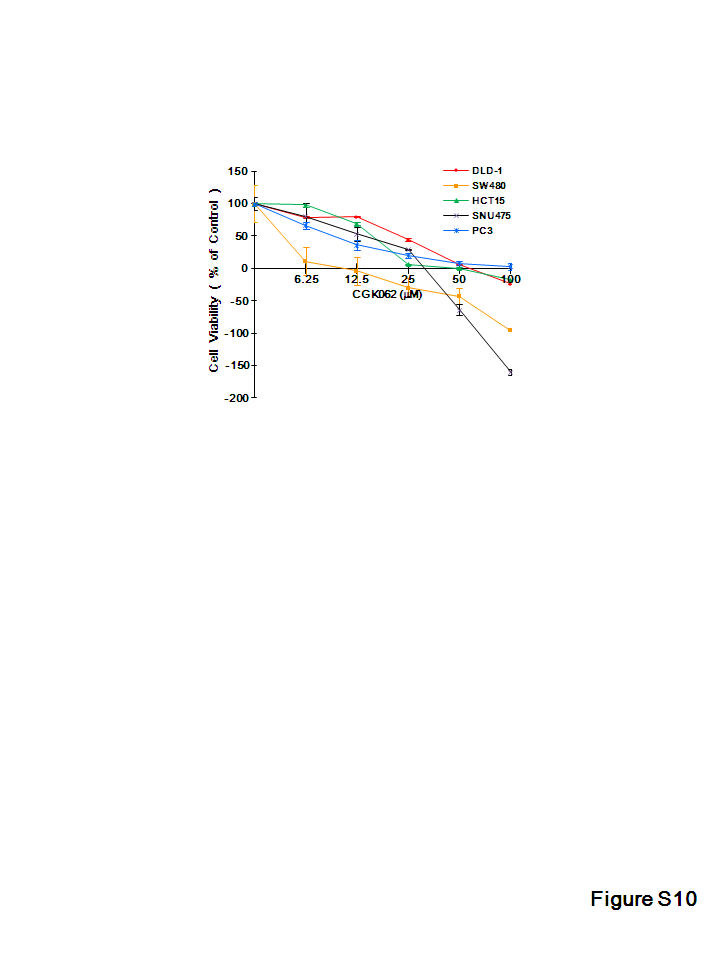

Supplement: Figure S10 — CGK062 inhibits the proliferation of CRT-positive cells. Cells were incubated, in the indicated concentrations of CGK062, for 48 hrs in 96-well plates. Cell viability was examined using the CellTiter-Glo assay (Promega). To calculate the inhibition of growth, the value at time 0 was subtracted. (TIF) [file pone.0046697.s010.tif]
